# Prevalence of stress, burnout, and job satisfaction among mental healthcare professionals in Jeddah, Saudi Arabia

**DOI:** 10.1371/journal.pone.0267578

**Published:** 2022-04-27

**Authors:** Turki Alqarni, Abdulrahman Alghamdi, Alhussain Alzahrani, Khalid Abumelha, Zahid Alqurashi, Ahmad Alsaleh

**Affiliations:** 1 College of Medicine, King Saud Bin Abdulaziz University for Health Sciences, Jeddah, Saudi Arabia; 2 King Abdullah International Medical Research Center, Jeddah, Saudi Arabia; 3 Psychiatry Section, Department of Medicine, King Abdulaziz Medical City, Ministry of the National Guard–Health Affairs, Jeddah, Saudi Arabia; 4 Department of Family Medicine, King Abdulaziz Medical City, Ministry of the National Guard–Health Affairs, Jeddah, Saudi Arabia; 5 Department of Medicine, King Faisal Specialist Hospital and Research Center, Jeddah, Saudi Arabia; 6 Department of Psychiatry, King Fahad Armed Forces Hospital, Ministry of Defence, Jeddah, Saudi Arabia; Shahrood University of Medical Sciences, ISLAMIC REPUBLIC OF IRAN

## Abstract

**Objective:**

To assess the levels of stress, burnout, and job satisfaction among mental healthcare professionals in Jeddah City, Saudi Arabia.

**Methods:**

A cross-sectional study was conducted on mental healthcare professionals in Jeddah between January 2017 and October 2018. Sociodemographic characteristics and levels of stress, burnout, and job satisfaction were assessed using the Perceived Stress Scale-14 (PSS-14), Maslach Burnout Inventory (MBI), and Job Satisfaction Scale (JSS). Descriptive statistics were used. Independent sample t-test, one-way ANOVA, Mann-Whitney, and Kruskal-Wallis tests were conducted to assess for effects of demographic variables on the perceived stress score, emotional exhaustion (EE) score, depersonalization (DP) score, professional accomplishment (PA) score, and the job satisfaction score (JSS).

**Results:**

A total of 107 participants were included (50.5% men; 49.5% women) with response rate of 79.2%. Prevalence of stress was 56.1%. High levels of emotional exhaustion and depersonalization were present among 41 (38.3%) and 26 (24.3%) of the respondents, respectively, while high score of low personal accomplishment were present among 61 (57%) respondents. In terms of job satisfaction, 25 (23.4%) were satisfied and 74 (69.2%) were indecisive. Male participants’ emotional exhaustion score (27±12) was significantly higher than females (22 ±10), (t(105) = 1.99, p-value = 0.049). Also, participants with a monthly income above SR 20,000 had significantly higher total job satisfaction (p-value = 0.041).

**Conclusions:**

Our findings suggest rates of stress and burnout among mental health professionals that warrant attention, with less than one-quarter of the participants being satisfied with their jobs. Further studies are needed to expand the findings and to explore the contributing factors. Additionally, interventions should be established by authorities to address the increasing rates of stress and burnout.

## Introduction

Increased awareness about the prevalence of mental distress in the healthcare setting has revealed considerable levels of occupational stress among healthcare professionals. When the stress persists for a prolonged duration, it eventually leads to burnout that evolves gradually and is described as a combination of emotional exhaustion, depersonalization, and low personal accomplishment [1].

The consequences of burnout are detrimental to healthcare services. It contributes to lower job satisfaction, decreased productivity, increased errors, poor patient care and a higher turnover [2, 3]. The harms also extend to economy. For example, burnout costs the healthcare system in the Unites States (US) around $4.6 billion dollars a year [4].

There are numerous studies that have examined stress and burnout among different specialties. The prevalence of burnout among the US physicians was found to be 54%, which was almost twice the prevalence among the general working staff [5]. Among nurses worldwide, a meta-analysis showed prevalence of 11.23% for burnout symptoms [6]. In Saudi Arabia, a cross-sectional study that was conducted on 582 consultants of different specialties revealed that majority of them experienced moderate to high levels of stress [7].

Adding to all previous literature on burnout among healthcare workers, the COVID-19 pandemic has imposed a greater deal of stress on them. A systematic review on all studies assessing burnout during the first year of the pandemic showed general trend of worsening in burnout rates compared to pre-pandemic rates [8]. A national study in Saudi Arabia between June and August, 2020, showed a substantial increase in burnout rates reaching 75% of the sample [9]. This upsurge was mainly associated with working for extra hours during the crisis, the feeling of being enforced to deal with COVID-19 patients, and being tested for COVID-19 multiple times [9]. In addition to the factors that contribute to stress and burnout in healthcare providers, mental health professionals may have additional factors such as the stigma attached to the mental health, exposure to negative emotions or traumatic experiences, dealing with unstable patients or patients with suicidal thoughts, and the long time spent in documentation [10]. Studies on prevalence of stress and burnout among mental health professional showed variable results. For instance, a study was conducted in Singapore showed that healthcare professionals who worked as part of a mental health setting had a higher level of stress which increased noticeably depending on demographic status, such as younger age, less experience, and low annual income [11]. Another study one North American psychiatrists showed that 78% amongst them suffered high levels of burnout [12]. A large systematic review on 62 studies that assessed burnout among mental healthcare professionals found that the average mental healthcare provider experienced moderate level of depersonalization and high level of emotional exhaustion but were able to maintain high level of personal accomplishment [10].

To our knowledge, no study in Saudi Arabia has examined mental wellbeing of all professionals at psychiatry departments and hospitals. Therefore, this study aimed to measure the prevalence of stress, burnout, and to assess job satisfaction among different mental healthcare professionals in Jeddah city, Saudi Arabia.

## Methods

This cross-sectional study was conducted between January 2017 and October 2018 in Jeddah, Saudi Arabia. In Jeddah, there are five psychiatry departments in general hospitals, one hospital for addiction, and one mental health hospital. The study population consisted of all psychiatrists, psychiatric residents, psychologists, social workers, and psychiatric nurses, men and women, across all age spectrum, who were working in these centers during the duration of the study. Professionals who did not speak English were excluded. A self-administered printed questionnaire was distributed to all 135 mental healthcare professionals who met the inclusion criteria. Ethical approval was obtained from the institutional review board at King Abdullah International Medical Research Centre. Informed consents were obtained from all participants.

The questionnaire consisted of a section addressing sociodemographic variables (e.g., gender, age, nationality, marital status, occupation, qualifications, number of working years, and income) followed by three widely used scales to measure perceived stress, burnout and job satisfaction. These scales were the Perceived Stress Scale (PSS), Maslach Burnout Inventory–Human Services Survey (MBI-HSS), and Job Satisfaction Scale (JSS) [13–15].

The PSS is a 14-item scale used to determine the level of stress that professionals experience. Items are rated on a 5-point scale, ranging from 0 (never) to 4 (very often). An item example includes: “in the last month, how often had you felt that you were not able to control the important things in your life?” The mean score was calculated and participants with higher score than average were labelled to be stressed.

The MBI-HSS is a 22-item scale to measure emotional exhaustion, depersonalization, and professional accomplishment. It was used to assess the level of burnout of participants. Items are rated on a 7-point scale, ranging from 0 (never) to 6 (everyday). An item example includes: “How often: working with people all day is really a strain for me”. For emotional exhaustion, A score of ≤ 16 and signified low levels of burnout, between 17 and 26 were considered as moderate burnout, and scores over 27 were of high-level burnout. Regarding depersonalization, the score of ≤ 6 or less indicated low levels of burnout, scores between of 7 and 12 were considered as moderate burnout and a score over 13 was of a high-level burnout. Lastly, personal accomplishment scores of ≤ 31 were a marker of high burnout, 38 to 32 indicated moderate burnout, and over 39 indicated low levels of burnout.

The JSS is a 36-item scale with nine subscales that are pay, promotion, supervision, fringe benefits, contingent rewards, operating conditions, coworkers, nature of work and communication. Items are rated on a 6-point scale, ranging from 1 (disagree very much) to 6 (agree very much). An Item example includes: “I feel a sense of pride in doing my job”. The absolute approach was used to compute and interpret the overall JSS score [16]. The ranges were 36 to 108 for dissatisfaction, 144 to 216 for satisfaction, and between 108 and 144 for indecisiveness.

All data were entered into an excel file and then were managed through Statistical Package for Social Sciences (SPSS) version 23. Descriptive statistical analyses were used. Values were reported as proportions and percentages for categorical variables and as means and standard deviations or modes with ranges for continuous variables. Since the PSS-14 has no cut-off value, the scores were stratified into four groups based on the quartiles, where the upper two combined were labelled as stressed and the lower two as not stressed. Independent sample t-test, one-way ANOVA, Mann-Whitney, and Kruskal-Wallis tests were conducted to assess for effects of demographic variables on the perceived stress score, emotional exhaustion score, depersonalization score, professional accomplishment, and the total job satisfaction score. Statistical significance was considered at p-value < 0.05.

## Results

A total 135 professionals were invited to participate, while 107 agreed to take part (response rate = 79.3%). Out of them, 54 (50.5%) participants were men. Majority of the sample were in the age group 20–35 years (70; 65%) and married (73; 68.2%). Professionals from other nationalities than Saudis represented only 18.7%. Physicians and physicians in-training were the main group of participants (52; 48.6%). Rest of the sociodemographic variables are shown in Table 1.

**Table 1:** Sociodemographic characteristics of the participants.

Variable		N	%
Gender	Male	54	50.5
	Female	53	49.5
Age	20–35	70	65.4
	36–50	33	30.8
	> 50	4	3.7
Nationality	Saudi	87	81.3
	Non-Saudi	20	18.7
Martial statues	Married	73	68.2
	Single	27	25.2
	Divorced/separated	7	6.5
Occupation	Psychiatrist	26	24.3
	Psychologist	18	16.8
	Social Worker	6	5.6
	Nurse	28	26.2
	Psychiatry Resident	26	24.3
	others	3	2.8
Qualification	Bachelor	55	51.4
	Postgraduate Degree	52	48.6
Years of employment	2 years or less	27	25.2
	3–6 years	41	38.3
	7–10 years	15	14.0
	more than 10 years	24	22.4
Monthly income (Saudi Riyals)	< 10,000	12	11.2
	10,000–19,999	67	62.6
	≥ 20,000	28	26.2

The overall mean PSS score was 25.9 (± 5.5). A total of 60 participants (56.1%) had high scores and were labelled stressed, accordingly (Table 2). Nurses were the most common subgroup of the participants to experience stress (18; 30%), followed by psychiatry resident (16; 26.7%).

**Table 2:** Stress prevalence among the participants.

Group	N	%
Non-stressed	47	43.9
Stressed	60	56.1
Overall Mean PSS-14 score	25.94 (± 5.47)	

In terms of burnout (Table 3), most of the participants had moderate emotional exhaustion (43; 40.2%) and depersonalization (46; 43%), and around 57% had lack of personal accomplishment. High levels of emotional exhaustion and depersonalization were present among 41 (38.3%) and 26 (24.3%) of the respondents, respectively.

**Table 3:** Burnout characteristics among the participants.

Burnout subscale		N	%
Emotional Exhaustion	Low	23	21.5
	Moderate	43	40.2
	High	41	38.3
Depersonalization	Low	35	32.7
	Moderate	46	43.0
	High	26	24.3
Personal accomplishment	Low	61	57.0
	moderate	26	24.3
	High	20	18.7

Of the 107 participants, 25 (23.4%) were overall satisfied about their job, while the majority were indecisive (74; 69.2%). Most of the sample were satisfied about the supervision (68; 63.6%) and nature of work (69; 64.5%). In contrast, more than half of the respondents (58; 54.2%) were dissatisfied about the operating conditions (Table 4).

**Table 4:** Satisfaction of the sample about their jobs.

Job satisfaction survey subscale	Satisfied	Dissatisfied	Indecisive
	N (%)		
Pay	38 (35.5)	31 (29)	29.0
Promotion	30 (28)	31 (29)	46 (43)
Supervision	68 (63.6)	15 (14)	24 (22.4)
Fringe Benefits	27 (25.2)	31 (29)	49 (45.8)
Contingent Rewards	36 (33.6)	34 (31.8)	37 (34.6)
Operating Conditions	58 (54.2)	19 (17.8)	30 (28)
Coworkers	11 (10.3)	54 (50.5)	42 (39.3)
Nature of Work	69 (64.5)	12 (11.2)	26 (24.3)
Communication	39 (36.4)	21 (19.6)	47 (43.9)
Total satisfaction score	25 (23.4)	8 (7.5)	74 (69.2)

As shown in Table 5, male participants’ emotional exhaustion score (27± 12) was significantly higher than females (22 ±10), (t(105) = 1.99, p-value = 0.049). Total job satisfaction score differed significantly among the three groups of the monthly income (p-value = 0.041). On post-hoc analysis, participants with a monthly income of 20,000 SR or above had significantly higher total job satisfaction score in comparison to those receiving from 10,000 to 19,999 SR (U = 629, p = 0.012). No other significant associations were found between stress, burnout and job satisfaction and socio-demographic factors.

**Table 5:** Association between sociodemographic variables and stress, burnout and job satisfaction scores.

Independent variables		Perceived stress score		Burnout						JSS	
				Emotional exhaustion		Depersonalization		Professional Accomplishment			
		Mean (±SD)	P-value	Mean (±SD)	P-value	Median (IQR)	P-value	Mean (±SD)	P-value	Median (IQR)	P-value
Sex	Male	26 (±5)	0.483*	27 (±12)	**0.049** *	11 (7)	0.064†	28 (±10)	0.142*	130 (18)	0.197†
	Female	26 (±6)		22 (±10)		9 (8)		30 (±9)		134 (22)	
Age	20–35	26 (±5)	0.249**	24 (±12)	0.996**	10 (9)	0.307††	29 (±10)	0.324**	131 (18)	0.542††
	36–50	25 (±6)		25 (±12)		9 (6)		30 (±9)		137 (39)	
	> 50	28 (±7)		24 (±7)		13 (11)		23 (±10)		133 (13)	
Nationality	Saudi	26 (±5)	0.818*	25 (±11)	0.396*	10 (6)	0.724†	29 (±9)	0.235*	131 (21)	0.408††
	Non-Saudi	26 (±6)		22 (±13)		10 (13)		31 (±9)		137 (22)	
Marital status	Married	26 (±6)	0.704**	25 (±11)	0.477**	10 (8)	0.386††	29 (±9)	0.472**	131 (22)	0.793††
	Single	27 (±6)		23 (±12)		9 (9)		29 (±11)		132 (14)	
	Divorced/separated	27 (±6)		22 (±8)		10 (8)		33 (±7)		142 (27)	
	Psychiatrist	25 (±4)	0.781**	25 (±9)	0.692**	10 (7)	0.591††	30 (±8)	0.843**	136 (17)	0.345††
	Psychologist	27 (±7)		24 (±11)		10 (11)		29 (±10)		132 (17)	
Occupation	Social Worker	24 (±4)		31 (±14)		14 (7)		32 (±5)		141 (17)	
	Nurse	26 (±7)		23 (±12)		9 (6)		29 (±9)		130 (21)	
	Psychiatry Resident	26 (±5)		24 (±13)		10 (10)		27 (±11)		128 (32)	
	others	26 (±5)		24 (±6)		12 (NA)		32 (±6)		145 (NA)	
qualification	Bachelor	26 (±6)	0.919*	23 (±12)	0.346*	9 (9)	0.518†	30 (±10)	0.536*	130 (19)	0.432††
	Postgraduate Degree	26 (±4)		26 (±11)		10 (6)		29 (±8)		135 (22)	
Years of employment	2 years or less	26 (±5)	0.304**	21 (±10)	0.090**	9 (8)	0.317††	29 (±11)	0.835**	130 (30)	0.521††
	3–6 years	27 (±6)		27 (±12)		11 (10)		29 (±8)		131 (21)	
	7–10 years	26 (±6)		26 (±10)		10 (6)		31 (±10)		131 (14)	
	more than 10 years	24 (±5)		22 (±12)		10 (8)		28 (±10)		138 (24)	
Monthly income (In Saudi Riyals)	< 10,000 SR	26 (±6)	0.276**	23 (±15)	0.452**	9 (11)	0.607††	31 (±10)	0.538**	132 (31)	**0.041** ††
	10,000–19,999	27 (±5)		24 (±11)		10 (8)		28 (±10)		129 (20)	
	≥ 20,000	25 (±6)		27 (±11)		10 (8)		30 (±9)		139 (20)	

* Independent sample T test.

** One way ANOVA.

† Mann-Whitney’s test.

†† Kruskal-Wallis’s test.

## Discussion

To our knowledge, our study is the first in Saudi Arabia to examine stress, burnout, and job satisfaction among mental healthcare professionals. Majority of the participants had a higher level of stress than the average, moderate to high prevalence of burnout symptoms and ambivalent job satisfaction.

Nurses were the main subgroup to suffer from stress. This goes in line with much research that showed mental health nurses to experience a great deal of stress [17, 18]. A longitudinal study on mental healthcare workers in the United Kingdom found also that social workers and nurses reported higher rates of emotional exhaustion with lower job satisfaction when compared to psychologists [19]. A possible explanation might be due longer working hours and closer work with patients. Resident physicians were close to nurses in prevalence of stress, with more than one forth of them being stressed. A previous study on stress among resident physicians in Saudi Arabia showed their mean PSS score to be higher than their peers in many other countries [20]. A systematic review of burnout studies that included 2,619 psychiatry resident reported an overall high burnout rate of 33.7%, but was falling in the range of burnout amongst residents in different specialties [21].

A systematic review and meta-analysis on burnout among mental health staff was conducted in 2018 and included 33 studies (n = 9409) [22]. The pooled prevalence of emotional exhaustion was estimated to 40% (CI 31%-48%), while that for depersonalization was around 22% (CI 15%-29%). The prevalence of low personal accomplishment was 19% (CI 13%-25%). Our findings were similar to these international figures with prevalence for emotional exhaustion (38.3%), and for depersonalization (24.3%). An exception to this was the sense of personal accomplishment dimension where more than have of the participants in our study had it low. This can be due to the fact that most of our sample fell in age group 20–35, and increasing age has been associated with increased sense professional accomplishment [23–26]. This finding is also affirmed by another study conducted on early career psychiatrist that showed high rates (39.9%) of low personal accomplishment [27].

Regarding the job satisfaction, our results indicated that 69.2% of the mental healthcare professionals were neither satisfied or dissatisfied, 23.4% were satisfied, and only 7.5% were not satisfied. Moreover, the healthcare professionals satisfaction had significant association with their income. This is similar to a study in a tertiary hospital in Saudi Arabia where satisfaction with income among physicians predicted total satisfaction about their jobs [28]. Also, larger scale studies internationally have reported that income satisfaction was positively associated with total job satisfaction, such as Leigh’s study on 12,474 physicians in the US [29].

Our findings shed the light on the increasing prevalence of the mental distress among mental healthcare workers in Jeddah city, Saudi Arabia. Interventions should be designed to identify the mental healthcare workers needs and to address them accordingly. A few interventional studies on mental healthcare workers have tried with some success to reduce the burden of burnout [30, 31]. For example, a total of 84 mental healthcare workers in were given a one-day session on how to reduce burnout [28]. A follow up survey six week later showed significant reduction in emotional exhaustion, depersonalization, and improvement in optimism.

This study has some limitations to be acknowledged. First, we excluded non-English speakers because the JSS had no validated Arabic version. Second, the study was conducted in one city and in cross-sectional design which limit its generalizability to all settings in Saudi Arabia. Third, some professions were underrepresented in our sample, such as social workers, which may also limit the generalizability of the results to this sub-group.

## Conclusion

Our findings suggest high rates of stress and burnout with less than one-quarter of the participants being satisfied about their jobs. Future large-scale research should be conducted on mental healthcare workers in other regions of Saudi Arabia for comparison and expansion of our findings. Interventions, such as educational sessions on stress and burnout, should be established by authorities to increasing rates of stress among mental health providers, explore the contributing factors, and to promote a better working environment for them. Also, further studies are needed explore other contributing factors that we did not look at.

## List of abbreviations

PSS-14: Perceived Stress Scale-14
MBI: Maslach Burnout Inventory
JSS: Job Satisfaction Scale
EE: emotional exhaustion score
DP: depersonalization score
PA: professional accomplishment score
JSS: job satisfaction score
US: Unites States
MBI: HSS, Maslach Burnout Inventory–Human Services Survey
SPSS: Statistical Package for Social Sciences
